# Flours from Swedish pulses: Effects of treatment on functional properties and nutrient content

**DOI:** 10.1002/fsn3.1280

**Published:** 2019-11-20

**Authors:** Ferawati Ferawati, Mohammed Hefni, Cornelia Witthöft

**Affiliations:** ^1^ Department of Chemistry and Biomedical Sciences Linnaeus University Kalmar Sweden; ^2^ Food Industries Department Faculty of Agriculture Mansoura University Mansoura Egypt

**Keywords:** dietary fiber, flour, folate, functional properties, pulses

## Abstract

Despite the high nutritional profile in pulses, pulse consumption in Sweden is still low. However, the recent increase in consumption of sustainable and locally produced food in Sweden is driving demand for a versatile, functional pulse‐based ingredient that can be incorporated into different food products. This study assessed different treatments (boiling, roasting, and germination) when preparing flour from domestically grown pulses (yellow pea, gray pea, faba bean, and white bean). Functional properties (water and oil absorption capacity, emulsion and foaming properties, and gelation concentration) of the flours produced following different treatments and their nutrient content (total dietary fiber, total choline, and folate content) were determined. Depending on pulse type, all treatments increased (*p* < .001) water absorption capacity up to threefold and gelation concentration up to twofold, whereas emulsion activity and foaming capacity decreased by 3%–33% and 5%–19%, respectively, compared with flour made from raw pulses. All treatments also had a significant effect (*p* < .001) on nutrient content. Total dietary fiber increased (*p* < .02) by 11%–33%, depending on treatment and pulse type. Boiling decreased (*p* < .001) total choline and folate content in all pulse flours, by 17%–27% and 15%–32%, respectively. Germination doubled folate content (*p* < .001) in flour from both pea types compared with flour from the raw peas. In conclusion, treated pulse flours could be useful in food applications such as coating batter, dressings, beverages, or bakery goods, to improve the content of fiber, total choline, and folate.

## Graphical abstract

Flours from domestically grown pulses in Sweden were prepared by using three treatments. Functional properties of the flours produced following different treatments and their nutrient content were determined. All treatments altered the functional properties and nutrient content of the pulse flours at different magnitudes, depending on the type of pulses. Flour from treated pulses could be utilized in food products such as coating batter, dressings, beverages, or bakery goods, to improve the content of fiber, total choline, and folate.

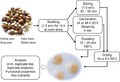

## 1 INTRODUCTION

Pulses are characterized by high contents of total dietary fiber and folate (FAO, 2016; Ramdath, Renwick, & Duncan, 2016), dietary intake of which is below the recommended level in Sweden (NFA, 2012). Pulse consumption in Sweden is low, only 1.9 kg/capita/year, compared with 4.8–5.6 kg/capita/year in Mediterranean countries, for example, Greece, Spain, and Italy (FAO, 2017). Long processing time, limited choice of pulse‐based commercial products, and presence of antinutritional compounds are commonly cited reasons for low consumption of pulses (Tosh et al., 2013). However, recent trends in Sweden for healthy and sustainable eating and for increased consumption of locally sourced foods have generated interest among food industries in using flour from domestically grown pulses to develop innovative and environmentally friendly food products (Olsson, 2017). Increased consumption of pulse‐based foods might improve the dietary fiber and folate intake of the Swedish population in the long term.

Pulse flour is a versatile component that can be incorporated into different food products. However, pulses also contain several antinutritional compounds, for instance, protease inhibitors hampering digestion of proteins, oligosaccharides that may cause flatulence and phytate that may chelate essential minerals thereby lowering their absorption (Sánchez‐Chino, Jiménez‐Martínez, Dávila‐Ortiz, Álvarez‐González, & Madrigal‐Bujaidar, 2015). Treatments such as soaking, wet or dry heating, and germination could reduce the content of antinutritional compounds and undesirable beany flavor in pulse flour (Jiang et al., 2016; Khattab & Arntfield, 2009). Furthermore, treatments could alter the functional properties and nutrient content (Aguilera, Esteban, Benítez, Mollá, & Martín‐Cabrejas, 2009). Water and oil absorption capacity, emulsion activity and stability, foaming capacity and stability, and gelation determine the performance of pulse flour as a food ingredient, thereby affecting the characteristics of the end‐product and consumer acceptance (Adebowale & Lawal, 2004). Accurate data on the content of nutrients such as dietary fiber, resistant starch, folate, and choline are needed for nutritional characterization of Swedish‐grown pulses.

Functional properties of flour from raw common beans, lentils, and chickpea have been investigated previously (Du, Jiang, Yu, & Jane, 2014; Kaur & Singh, 2005; Siddiq, Ravi, Harte, & Dolan, 2010). The effect of treatments on functional properties or nutrient content in particular types of pulses has also been investigated (Giami, 1993; Mang et al., 2015; Prinyawiwatkul, Beuchat, McWatters, & Phillips, 1997; Wang, Hatcher, & Gawalko, 2008). However, only a limited number of studies have systematically compared the effect of different treatments on the functional properties and nutrient content of flours made from pulses of interest such as yellow pea, gray pea, faba bean, and white bean that could be cultivated in Sweden. Additionally, to the best of our knowledge, no previous study has characterized flours made from raw or treated Swedish‐grown pulses.

The aim of this work was thus to study the effect of different treatments on the functional properties and content of nutrients in the flour from domestically grown pulses in Sweden.

## 2 MATERIALS AND METHODS

### 2.1 Materials

Dried yellow pea (*Pisum sativum* var. *clara*), gray pea (*Pisum sativum*, unknown Latvian variety), and white bean (*Phaseolus vulgaris* var. *T9905*) harvested in 2016 were obtained from Kalmar‐Ölands Trädgårdsprodukter (KÖTP), Sweden. Dried faba bean (*Vicia faba* var. *alexia*) harvested in 2017 was obtained from Nordisk Råvara, Sweden. These pulses were chosen based on their suitability for cultivation in Sweden. All pulses were stored in a box at room temperature until processing. All‐purpose white wheat flour (Kungsörnen, Lantmännen) purchased at a local supermarket in Kalmar, Sweden, was used as a reference.

### 2.2 Methods

#### 2.2.1 Preparation of pulse flour

Three treatments were chosen to process the Swedish pulses: roasting, boiling, and germination. Pulses were soaked at room temperature for 14 hr in tap water (1:3 w/v) before all treatments, and 200 g soaked pulses were used for each treatment. Pulse flour preparation was carried out in duplicate trials.

*Roasting*: Soaked pulses were roasted in an oven (HBA530B0S, Bosch) at 180°C for 15 min for yellow pea, 25 min for gray pea, and 20 min for both bean types (Xu et al., 2016) to moisture content <15%.

*Boiling*: Soaked pulses were boiled in water (1:5 w/v) until they were soft, for 50 min for yellow pea, 35 min for gray pea, 45 min for faba bean, and 30 min for white bean. The boiled pulses were dried in an oven (TS8024, Termaks) at 50 ± 1.5°C for 16 hr to moisture content <15%.

*Germination*: Soaked pulses were kept between layers of wet tissue paper and germinated in an incubator (INCU‐line, VWR) at 20°C for both, 24 hr and 48 hr. The germinated pulses were steamed in a sieve over boiling water for 4 min and then dried in an oven (TS8024, Termaks) at 50 ± 1.5°C for 8 hr to moisture content <15%.

The dried treated pulses were milled to 500 mm particle size using a laboratory‐scale mill (Cyclotec 1093, Tecator). Raw pulse flours were also prepared as control samples, by milling the raw pulse seeds. All samples were packed under vacuum in polyethylene bags and stored at −20°C until analysis.

#### 2.2.2 Analysis of functional properties

Functional properties were determined on duplicate samples of raw and treated pulse flours (*n* = 4, duplicate samples, duplicate trials). Analyses were repeated if the coefficient of variation (CV) for duplicate samples exceeded 15%.

For determination of water absorption capacity (Kaur & Singh, 2005), 3 g of sample was dispersed in 25 ml distilled water in a preweighed tube, stirred every 5 min for 30 min, and centrifuged at 3,000 *g* for 25 min (GS‐15, Beckman). The supernatant was discarded, excess moisture was removed by oven‐drying (TS8024, Termaks) at 50°C for 25 min, and the tube was reweighed. For determination of oil absorption capacity (Kaur & Singh, 2005), 0.5 g sample was dispersed in 6 ml corn oil in a preweighed tube, stirred for 1 min, left for 30 min, and centrifuged at 3,000 *g* for 25 min (GS‐15, Beckman). After centrifugation, the oil layer was discarded, and the tube was inverted to drain excess oil and then reweighed.

For determination of emulsion activity (Kaur & Singh, 2005), 0.875 g of sample was homogenized with 12.5 ml water at 10,000 rpm for 30 s (Polytron PT 3100, Kinematica). Peanut oil was added (6.25 ml), and the mixture was homogenized (10,000 rpm, 30 s). Another 6.25 ml of oil was added, and the mixture was homogenized again at the same speed for 90 s. The emulsion was transferred into two 15 ml centrifuge tubes and centrifuged at 1,100 *g* for 5 min (GS‐15, Beckman).

For determination of emulsion stability (Kaur & Singh, 2005), the samples were prepared as above. The emulsified samples in tubes were heated in a water bath (Grant OLS 200, Fisher Scientific) at 85°C for 15 min, cooled, and centrifuged at 1,100 *g* for 5 min (GS‐15, Beckman). Emulsion activity and stability was then calculated as:

(1) $$\text{Emulsion}\;\text{activity}\;\text{and}\;\text{stability}\;\left(\text{EA,}\;\text{ES,}\;\%\right)=\frac{\text{Volume}\;\text{of}\;\text{emulsified}\;\text{layer}}{\text{Total}\;\text{volume}\;\text{of}\;\text{emulsion}}\times 100$$

For determination of foaming properties (Kaur & Singh, 2005), 1.5 g of sample was homogenized with 50 ml distilled water at 10,000 rpm for 2–3 min (Polytron PT 3100, Kinematica). The mixture was immediately transferred to a measuring cylinder, and 10 ml distilled water was used for rinsing and added to the measuring cylinder. Foam volume in the measuring cylinder was recorded at intervals of 20, 40, 60, and 120 min. Foaming capacity was then calculated as

(2) $$\text{Foaming}\;\text{capacity}\;\left(\text{FC, \textbackslash{}\%}\right)=\frac{\text{Volume}\;\text{after}\;\text{whipping}-\text{Volume}\;\text{before}\;\text{whipping}\;}{\text{Volume}\;\text{before}\;\text{whipping}}\times 100$$

and foaming stability was determined as

(3) $$\text{Foaming}\;\text{stability}\;\left(\text{FS, \textbackslash{}\%}\right)=\frac{\text{Volume}\;\text{of}\;\text{foam}\;\text{after}\;t\left(t=20;40;60;120\;\min\right)}{\text{Initial}\;\text{foam}\;\text{volume}}\times 100$$

For determination of least gelation concentration (Sathe, Deshpande, & Salunkhe, 1982), suspensions of pulse flour at concentrations of 2, 4, 6, 8, 10, 12, 14, 16, 18, and 20% (w/v) in 5 ml distilled water were prepared, heated in a boiling water bath for 1 hr, and cooled at 4°C for 2 hr. The least gelation concentration was established as that at which the sample did not fall out when the test tube was inverted.

#### 2.2.3 Nutrient analysis

All nutrient analyses were performed on duplicate samples of raw and treated pulse flour from each flour preparation trial (*n* = 4, duplicate samples, duplicate trials).

The resistant starch (RS) content was measured according to AOAC 2002.02 (AOAC, 2000a), using the K‐RSTAR kit from Megazyme, Ireland. In brief, 0.1 g sample and 4 ml enzyme solution (α‐amylase and amyloglucosidase in sodium maleate buffer) were vortexed and incubated in a shaking water bath at 37°C for 16 hr. Thereafter, 4 ml ethanol (99% v/v) was added to stop the hydrolysis, and the mixture was centrifuged (1,500 *g*, 10 min). The pellet was resuspended twice with 50% ethanol and centrifuged again. The pellet was redissolved in 2 ml 2 M KOH to solubilize the RS. The sample was neutralized with 8 ml acetate buffer, and 100 µl amyloglucosidase was added to hydrolyze the starch to glucose during incubation at 50°C for 30 min. Next, 3 ml glucose oxidase–peroxidase–aminoantipyrine (GOPOD) reagent was added to 100 µl of the mixture and incubated at 50°C for 20 min. The glucose content was determined at 510 nm (Ultrospec 3000, Pharmacia Biotech).

The total dietary fiber (TDF) content was measured according to AOAC 991.43 (AOAC, 1995), using the K‐TDFR kit from Megazyme, Ireland. In brief, 1 g of sample and 15 ml MES/TRIS buffer pH 8.2 containing 50 µl thermostable α‐amylase were incubated in a boiling water bath for 30 min. After cooling, 100 µl protease solution was added, and the mixture was incubated at 60°C for 30 min. After pH adjustment to 4.1–4.8 with 5 ml 0.561 N HCl, 100 µl amyloglucosidase was added and the mixture was incubated again at 60°C for 30 min. Ethanol 95% (225 ml) was added to precipitate the fiber. The mixture was filtered, and the residue was washed with aqueous ethanol (78% and 95%) and dried. Crude protein content (using Kjeldahl method) and ash content (by ashing at 525°C) were determined on the residue to correct the TDF value.

Total choline was quantified according to Hefni, Schaller, and Witthöft (2018) by HPLC‐FLD (Agilent 1260, Agilent Technologies). In brief, 0.25 g of sample was incubated in 10 ml 1 M HCl at 60°C for 18 hr, cooled at room temperature, neutralized with NaOH, and centrifuged (2,600 *g*, 15 min). The supernatant was filtered with a 0.22 µm PES syringe filter, and 1 ml acetonitrile and 80 mg magnesium oxide were added to 20 µl of extract and vortexed. Then, 60 µl NaOH 1 M and 20 µl 1‐naphthyl isocyanate were added, and the mixture was shaken at room temperature for 15 min. Next, 60 µl water was added and the mixture was vortexed and centrifuged (13,000 *g*, 5 min). Total choline was quantified on a strong cation column (150 mm × 4.6 mm × 5 µm) (Phenomenex Luna) using HPLC‐FLD (Agilent 1260, Agilent Technologies). A multilevel external calibration curve (*n* = 6, *R* ^2^ = .9992) was used for quantification based on fluorescence detection (ex/em 220/350 nm).

Folate quantification was performed according to Hefni, Öhrvik, Tabekha, and Witthöft (2010) by tri‐enzyme treatment. In brief, ~0.8 g of sample, 15 ml phosphate buffer, and 60 µl thermostable α‐amylase were incubated in a boiling water bath for 12 min. After cooling, 0.8 ml protease was added and the mixture was incubated at 37°C for 1.5 hr, followed by boiling (5 min) to inactivate protease. After cooling again and centrifugation, the extract was diluted to 25 ml with extraction buffer. Then, 130 µl dialyzed rat serum and 40 µl α‐amylase were added to 4 ml diluted extract, and the mixture was incubated at 37°C for 2 hr. The final extract was purified using a strong anion exchange cartridge and quantified using HPLC‐UV/FLD. Due to difficulties in quantifying 5‐HCO‐H_4_folate, it was converted to H_4_folate according to Hefni et al. (2018). In brief, 1 ml of the purified extract and 0.5 ml concentrated HCl were incubated at room temperature for 20 min, NaBH_4_ solution was added to a total volume of 5 ml, and the sample incubated at room temperature for 60 min. The 5‐HCO‐H_4_folate content was obtained as the difference between H_4_folate after and before conversion. Folate was quantified on a C_18_‐PFP column (150 mm × 4.6 mm × 3 µm) with a C_18_ guard column (13 mm × 10 mm × 3 µm) (Chromtech ACE, Scantec Nordic) using HPLC‐UV/FLD (Agilent 1260, Agilent Technologies). A multilevel external calibration curve (*n* = 7, *R* ^2^ = .9997 for H_4_‐folate, 0.9999 for 5CH_3_‐H_4_folate, 0.9996 for 10‐HCO‐PteGlu) was used for quantification based on fluorescence detection (ex/em 290/360 nm for H_4_folate, 5‐CH_3_‐H_4_folate, and 5‐HCO‐H_4_folate and 360/460 nm for 10‐HCO‐PteGlu) and multi‐wavelength detector (290 nm for folic acid).

The moisture content was determined according to AOAC (2000b), by drying 2 g of sample in an oven at 105°C overnight until it reached constant weight.

Coefficient of variation (CV) for RS, TDF, total choline, and folate between duplicate samples and duplicate flour preparation trials was below 11% in all cases.

#### 2.2.4 Statistical analysis

All data were expressed on a dry matter (DM) basis as mean ± standard deviation (*SD*). Two‐way ANOVA and post hoc Tukey's test were used to determine significant differences in water and oil adsorption capacity, emulsion activity and stability, foaming capacity and stability, least gelation capacity, RS, TDF, total choline content, and folate content, as a function of type of treatment and type of pulse. The level of significance was set at *p* < .05. All statistical analyses were performed using R software version 3. 4. 1‐2017.

## 3 RESULTS AND DISCUSSION

### 3.1 Functional properties

**Figure 1:**
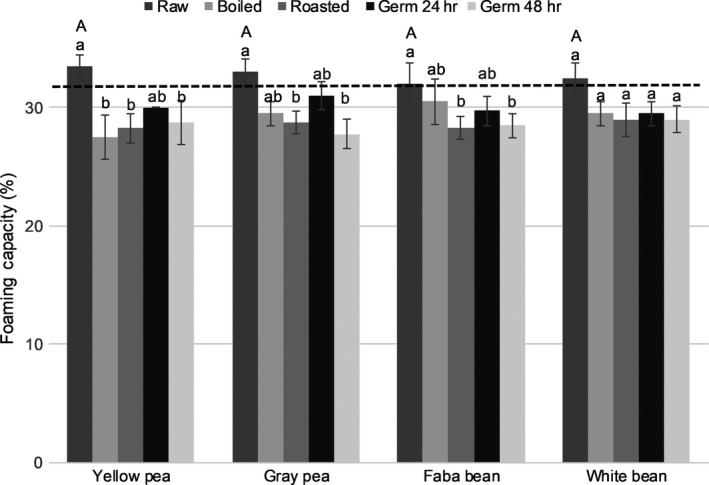
Foaming capacity of flours made from raw and treated pulses in 3 g/100 ml suspension. Bars represent the mean ± *SD* of duplicate samples from duplicate trials (*n* = 4). Means within each type of pulse with different letters are significantly different (Tukey's test, *p* < .05). For raw flours, means with different capital letters are significantly different (Tukey's test, *p* < .05). The dashed line (**‐‐**) shows the foaming capacity of reference wheat flour (*n* = 4, analysis in duplicate of two samples)

**Figure 2:**
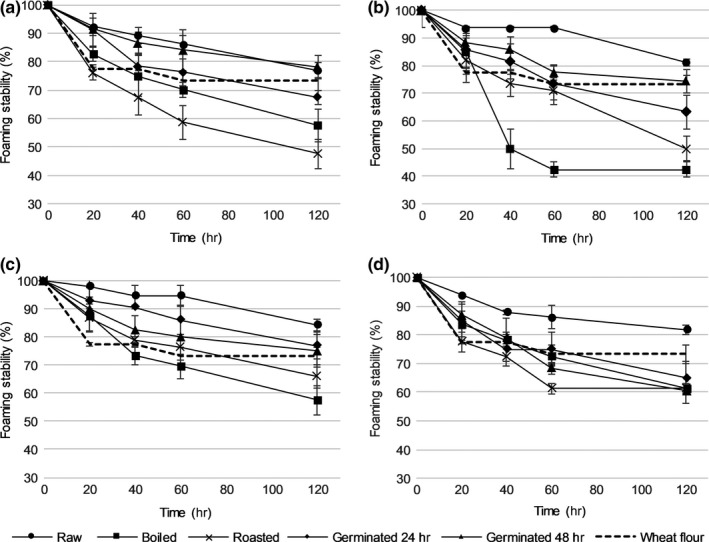
Foaming stability of flours made from raw and treated (a) yellow pea, (b) gray pea, (c) faba bean, and (d) white bean in 3 g/100 ml suspension. Each data point represents the mean of duplicate samples from duplicate trials (*n* = 4). Error bars: standard deviation. The dashed line (**‐‐**) shows foaming stability of reference wheat flour (*n* = 4, analysis in duplicate of two samples)

**Figure 3:**
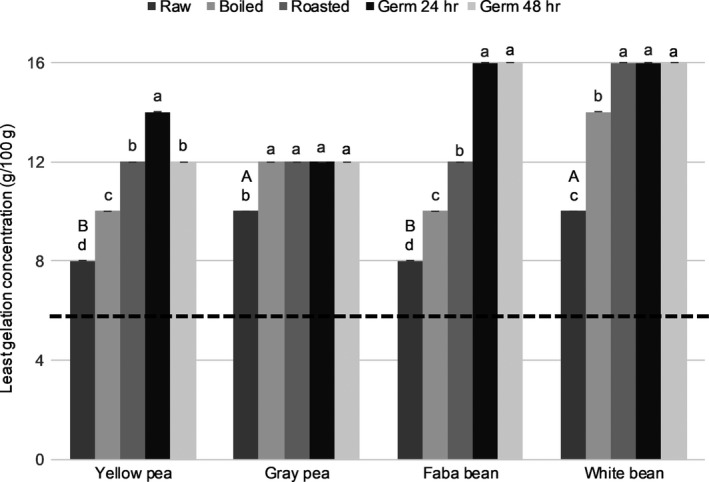
Least gelation capacity of flours made from raw and treated pulses. Bars represent the mean ± *SD* of duplicate samples from duplicate trials (*n* = 4). Means within each type of pulse with different letters are significantly different (Tukey's test, *p* < .05). For raw flours, means with different capital letters are significantly different (Tukey's test, *p* < .05). The dashed line (**‐‐**) shows least gelation capacity of reference wheat flour (*n* = 4, analysis in duplicate of two samples)

All flours from raw pulses (yellow pea, gray pea, faba bean, and white bean) had similar oil absorption capacity (OAC, *p* > .37), emulsion activity (EA, *p* > .99), emulsion stability (ES, *p* > .99) (Table 1), foaming capacity (FC, *p* > .99), and foaming stability (FS, *p* > .87) (Figures 1 and 2). However, raw yellow pea flour had lower (*p* < .001) water absorption capacity (WAC) than flours made from other raw pulses (Table 1). Flours from raw yellow pea and raw faba bean had a similar least gelation concentration (LGC) value, around 8%, while flour from raw gray pea and raw white bean had a higher LGC value (10%) (*p* < .001) (Figure 3).

**Table 1:** Water absorption capacity (WAC), oil absorption capacity (OAC), emulsion activity (EA), and emulsion stability (ES) of flours made from raw and treated pulses† and of wheat flour (for reference)‡

Flour	Raw	Boiled	Roasted	Germ 24 hr	Germ 48 hr
WAC (g water/g DM)					
Yellow pea	0.8 ± 0.03^cB^	1.9 ± 0.09^b^	2.4 ± 0.01^a^	1.9 ± 0.07^b^	2.2 ± 0.15^a^
Gray pea	1.1 ± 0.04^dA^	2.0 ± 0.01^c^	2.6 ± 0.22^a^	2.3 ± 0.16^b^	2.5 ± 0.04^ab^
Faba bean	1.1 ± 0.01^cA^	2.0 ± 0.02^b^	2.9 ± 0.12^a^	2.1 ± 0.06^b^	2.1 ± 0.09^b^
White bean	1.2 ± 0.02^cA^	2.1 ± 0.00^a^	2.2 ± 0.07^a^	1.9 ± 0.00^b^	1.9 ± 0.04^ab^
Wheat flour	0.7 ± 0.04				
OAC (g oil/g DM)					
Yellow pea	0.9 ± 0.05^bA^	0.8 ± 0.01^c^	1.0 ± 0.08^ab^	1.1 ± 0.00^a^	1.1 ± 0.02^a^
Gray pea	1.0 ± 0.03^aA^	0.8 ± 0.03^b^	1.0 ± 0.04^a^	1.1 ± 0.04^a^	1.1 ± 0.01^a^
Faba bean	1.0 ± 0.02^aA^	0.7 ± 0.04^b^	1.0 ± 0.01^a^	1.0 ± 0.01^a^	1.0 ± 0.01^a^
White bean	1.0 ± 0.04^aA^	0.8 ± 0.02^b^	1.1 ± 0.03^a^	1.1 ± 0.04^a^	1.0 ± 0.02^a^
Wheat flour	0.9 ± 0.01				
EA (%)					
Yellow pea	49.6 ± 0.59^aA^	47.5 ± 0.59^ab^	42.9 ± 2.95^b^	50.0 ± 0.00^a^	46.5 ± 0.88^ab^
Gray pea	50.0 ± 0.00^aA^	45.2 ± 3.54^ab^	42.9 ± 2.95^b^	42.3 ± 5.01^b^	33.3 ± 2.95^c^
Faba bean	51.3 ± 0.00^aA^	49.6 ± 0.59^a^	47.5 ± 3.54^a^	46.5 ± 3.83^a^	47.7 ± 0.88^a^
White bean	50.0 ± 0.00^aA^	41.3 ± 0.59^b^	50.0 ± 0.00^a^	49.6 ± 0.59^a^	48.1 ± 0.29^a^
Wheat flour	12.1 ± 0.59				
ES (%)					
Yellow pea	50.0 ± 0.00^aA^	50.0 ± 0.00^a^	47.5 ± 2.36^a^	49.6 ± 0.59^a^	47.5 ± 0.59^a^
Gray pea	50.0 ± 0.00^aA^	45.8 ± 0.00^ab^	37.5 ± 2.95^c^	42.3 ± 5.01^b^	26.0 ± 1.47^d^
Faba bean	50.2 ± 1.47^aA^	44.6 ± 2.36^ab^	48.1 ± 1.47^ab^	42.5 ± 3.54^b^	40.2 ± 3.83^b^
White bean	51.7 ± 0.59^aA^	39.8 ± 1.47^b^	49.6 ± 0.59^a^	45.4 ± 0.59^ab^	42.9 ± 0.00^b^
Wheat flour	15.0 ± 1.18				

Note

Means within rows with different letters are significantly different (Tukey's test, *p* < .05). Regarding specific functional parameters in raw flours, means within columns with different capital letters are significantly different (Tukey's test, *p* < .05).

^†^ All values are mean ± *SD* of duplicate samples from duplicate trials (*n* = 4) ± *SD*.

^‡^ Analysis in duplicate of two samples of commercial wheat flour (*n* = 4).

All functional properties of flours from raw pulses analyzed in this study, except foaming, were within the ranges reported for flours from several types of pulses (raw pinto bean, black bean, black eye bean, small red bean, and pea) (Du et al., 2014; Kaur, Sandhu, & Singh, 2007; Siddiq et al., 2010). However, the same authors reported higher values for FC (35%–55%) and FS (80%–100%) than in the present study (FC 32%–34%; FS 77%–84%). Variation of foaming properties might, according to others (Adebowale & Lawal, 2004; Du et al., 2014), be attributed to the variable content of proteins and carbohydrates, which both might affect the foam formation and stability.

In general, all treatments affected (*p* < .001) the functional properties of the pulse flours. For example, WAC (Table 1) and LGC (Figure 3) increased in all flours made from treated pulses. For some of the treated pulse flours tested, there was a decrease in emulsion properties (Table 1) and foaming properties (Figures 1 and 2) compared with flours made from the raw pulses.

Boiling, roasting, and germination all induced an increase (*p* < .001) in WAC of between 1.5‐fold and threefold for the flours made from treated pulses compared with raw pulses (Table 1). Similar results have been reported by others for flours made from germinated jack bean and boiled cowpea (Benítez et al., 2013; Giami, 1993). All treatments also led to a 1.5‐fold to twofold increase (*p* < .001) in LGC in flours made from all pulse types (Figure 3). Likewise, an increase in LGC of up to 1.5‐fold in flour made from boiled cannellini bean and cowpea has been observed by others (Aguilera, Estrella, Benitez, Esteban, & Martín‐Cabrejas, 2011; Prinyawiwatkul et al., 1997).

There was an 11%–30% decrease (*p* < .001) in OAC after boiling, depending on the pulse type (Table 1). Similarly, others have reported a 33% decrease in OAC in flour from boiled lima bean compared with flour from the raw beans (Marisela, Yannellis, & Alexia, 2007). In the present study, roasting and pregerminating all types of pulses did not affect (*p* > .10) OAC compared with that in the flours made from the raw pulses (Table 1).

After boiling, EA was reduced (*p* < .001) by 17% and ES by 25% (*p* < .001) in white bean flour, but did not affect (*p* > .41) the emulsion properties of other pulse flours (Table 1). This confirms findings by others (Aguilera et al., 2011) of a reduction in EA in flour from cooked cannellini bean and pinto bean. In contrast, roasting led to a 14% decrease in EA (*p* < .008) in yellow pea and gray pea flour, and a 25% decrease in ES (*p* < .001) in gray pea flour (Table 1). The germination treatment reduced (*p* < .007) EA by 15%–33% in gray pea flour and ES by 14%–48% in gray pea, faba, and white bean flours, compared with the flours made from the raw pulses (Table 1). Flours from roasted gray pea and germinated gray pea had lower (*p* < .004) emulsion properties than flours from the other treated pulses (Table 1).

Boiling resulted in an 18% reduction (*p* < .001) in FC in yellow pea flour, but did not affect (*p* > .19) the FC of other pulse flours (Figure 1). Roasting and 48 hr germination reduced (*p* < .02) FC by 11%–16% in pea and faba bean flour compared with the flours made from the raw pulses (Figure 1). Other studies report a 20%–25% reduction in FC in flour made from cooked cannellini bean (Aguilera et al., 2011), findings which are confirmed by the present study. The FS in all treated pulse flours decreased by 21%–48% (*p* < .001) after boiling and roasting (Figure 2). Flours from boiled gray pea and roasted gray pea had lower (*p* < .001) FS than the other treated pulse flours (Figure 2). The present findings are consistent with others (Giami, 1993) who observed a reduction in FS (by 10%–23%) in flour made from cooked and germinated cowpea.

Functional properties are mainly affected by proteins (Farooq & Boye, 2011). All treatments in the present study involved a heating step, which may have led to denaturation of proteins. It has been suggested that denaturation exposes hydrophilic and hydrophobic parts of proteins, thus affecting the water and oil absorption capacity and gelation properties (Aguilera, Esteban, et al., 2009). Denatured proteins may also become insoluble, making them unable to position themselves on the oil–water interface to form a film, thereby reducing emulsion and foam formation and stability (Prinyawiwatkul et al., 1997). Similar mechanisms might have operated in the treated flours in the present study, resulting in alterations in the functional properties in pulse flours after processing.

Compared with the reference commercial wheat flour, all treated pulse flours had threefold to fourfold higher WAC, twofold to fourfold higher EA, twofold to threefold higher ES, and up to threefold higher LGC. The roasted and germinated pulse flours had similar OAC as compared with wheat flour. However, depending on the type of pulse, treatments altered foaming properties by lowering FC by 2%–12% and FS by 8%–42% as compared with wheat flour.

### 3.2 Nutrient content

Flours from raw beans had a higher nutrient content than flours from raw peas, for example, total dietary fiber (TDF) was 13%–16% higher (Table 2), and total choline and folate content were twofold to threefold higher (Table 3). The TDF content of flour from raw pulses determined in this study was in the range of 16–18 g/100 g DM. Similar content (15–22 g/100 g DM) was reported by others for flours from raw black turle, cranberry, and red kidney beans (Wang, Hatcher, Tyler, Toews, & Gawalko, 2010). The resistant starch (RS) content of the raw pulse flours varied, with a notably higher content in white bean (34 mg/100 g DM), that is, sixfold to 20‐fold higher than in yellow pea, gray pea, and faba bean (Table 2). Likewise, others report similar range of RS in raw pea (2–6 g/100 g) and beans (30–37 g/100 g) (Dostálová, Horáček, Hasalová, & Trojan, 2009; Wang et al., 2010).

**Table 2:** Content of **r**esistant starch (RS) and total dietary fiber (TDF) in flours made from raw and treated pulses† and in wheat flour (for reference)

Flour	Raw	Boiled	Roasted	Germ 24 hr	Germ 48 hr
RS (g/100 g DM)					
Yellow pea	2.5 ± 0.25^cC^	4.2 ± 0.33^a^	2.6 ± 0.14^c^	3.3 ± 0.18^b^	3.5 ± 0.00^b^
Gray pea	5.4 ± 0.14^aB^	4.4 ± 0.01^b^	2.5 ± 0.18^d^	3.5 ± 0.07^c^	3.5 ± 0.01^c^
Faba bean	1.4 ± 0.10^dD^	4.0 ± 0.39^a^	2.8 ± 0.06^c^	3.3 ± 0.07^b^	3.4 ± 0.04^b^
White bean	33.6 ± 0.59^aA^	4.5 ± 0.02^b^	2.8 ± 0.01^d^	3.6 ± 0.22^c^	3.6 ± 0.04^c^
Wheat flour	0.2–0.4‡				
TDF (g/100 g DM)					
Yellow pea	15.7 ± 0.48^cB^	21.4 ± 1.13^a^	15.3 ± 1.10^c^	16.4 ± 0.26^bc^	17.5 ± 1.06^b^
Gray pea	15.9 ± 1.51^bB^	20.8 ± 0.69^a^	19.7 ± 0.46^a^	19.9 ± 0.02^a^	20.3 ± 0.32^a^
Faba bean	18.3 ± 1.01^bA^	22.2 ± 0.26^a^	19.1 ± 0.62^b^	20.3 ± 0.54^ab^	22.4 ± 0.14^a^
White bean	18.0 ± 0.14^cA^	23.7 ± 0.53^a^	21.0 ± 0.24^b^	22.6 ± 0.54^ab^	22.4 ± 0.54^ab^
Wheat flour	3.6§				

Note

Means within rows with different letters are significantly different (Tukey's test, *p* < .05). Regarding specific nutrients in raw flours, means within columns with different capital letters are significantly different (Tukey's test, *p* < .05).

^†^ All values are mean ± *SD* of duplicate samples from duplicate trials (*n* = 4).

^‡^ Value from Dhital, Katawal, and Shrestha (2010).

^§^ Value from the Swedish National Food Agency (NFA, 2018).

**Table 3:** Content of total choline and sum of folate in flours made from raw and treated pulses† and in wheat flour (for reference)

Flour	Total choline (mg/100 g, DM)	Folate content (µg/100 g, DM)				
		H_4_folate	5‐CH_3_‐H_4_folate	10‐HCO‐PteGlu	5‐HCO‐H_4_folate‡	Sum of folate as folic acid§
Yellow pea						
Raw	136 ± 4.9^aB^	16 ± 0.8^bD^	13 ± 1.4^dC^	38 ± 0.4^abB^	11 ± 1.3^bC^	73 ± 2.9^dD^
Boiled	127 ± 7.7^a^	12 ± 1.5^c^	13 ± 0.3^d^	34 ± 1.5^b^	5 ± 0.6^c^	62 ± 3.7^e^
Roasted	143 ± 3.5^a^	22 ± 0.5^a^	19 ± 0.5^c^	44 ± 1.2^a^	11 ± 1.3^b^	92 ± 2.4^c^
Germinated 24 hr	131 ± 4.6^a^	21 ± 1.5^a^	70 ± 3.2^b^	42 ± 1.3^a^	16 ± 1.6^a^	142 ± 7.3^b^
Germinated 48 hr	125 ± 1.5^a^	22 ± 1.5^a^	184 ± 13.2^a^	40 ± 2.7^ab^	20 ± 0.1^a^	254 ± 11.5^a^
Gray pea						
Raw	141 ± 4.7^aB^	31 ± 2.6^cC^	12 ± 0.2^dC^	37 ± 3.6^aB^	14 ± 1.6^bC^	90 ± 0.5^dC^
Boiled	129 ± 2.2^a^	19 ± 1.9^d^	15 ± 0.0^c^	35 ± 0.4^a^	7 ± 0.5^d^	72 ± 1.9^e^
Roasted	142 ± 3.8^a^	39 ± 0.2^b^	16 ± 0.5^c^	41 ± 1.4^a^	10 ± 0.9^c^	102 ± 2.5^c^
Germinated 24 hr	134 ± 4.1^a^	42 ± 1.5^ab^	50 ± 5.1^b^	41 ± 2.9^a^	16 ± 0.2^ab^	144 ± 6.1^b^
Germinated 48 hr	141 ± 1.2^a^	48 ± 0.7^a^	160 ± 5.9^a^	40 ± 0.3^a^	19 ± 1.4^a^	256 ± 6.5^a^
Faba bean						
Raw	155 ± 7.2^aB^	44 ± 2.1^aB^	73 ± 2.1^bA^	61 ± 1.9^aA^	47 ± 5.7^aB^	215 ± 3.5^aB^
Boiled	129 ± 5.7^b^	14 ± 0.3^d^	105 ± 15^b^	55 ± 5.1^a^	12 ± 1.2^c^	178 ± 18.8^b^
Roasted	137 ± 5.8^a^	25 ± 0.4^b^	139 ± 0.4^a^	62 ± 1.1^a^	20 ± 1.6^b^	235 ± 2.5^a^
Germinated 24 hr	140 ± 5.1^a^	20 ± 1.3^c^	149 ± 8.1^a^	61 ± 1.3^a^	11 ± 1.2^c^	231 ± 9.1^a^
Germinated 48 hr	146 ± 2.9^a^	20 ± 0.2^c^	131 ± 3.3^a^	63 ± 1.7^a^	17 ± 1.0^b^	220 ± 0.4^a^
White bean						
Raw	209 ± 11.2^aA^	85 ± 2.3^aA^	34 ± 1.3^bB^	37 ± 0.2^aB^	101 ± 0.1^aA^	245 ± 1.2^bA^
Boiled	152 ± 3.7^c^	17 ± 0.8^d^	78 ± 2.5^b^	40 ± 0.9^a^	40 ± 3.8^c^	166 ± 5.9^c^
Roasted	214 ± 18.8^a^	40 ± 3.8^b^	126 ± 3.9^a^	42 ± 5.7^a^	88 ± 6.4^ab^	282 ± 0.7^a^
Germinated 24 hr	177 ± 11.7^b^	20 ± 2.2^cd^	130 ± 5.4^a^	42 ± 8.7^a^	78 ± 13.8^b^	256 ± 17.9^ab^
Germinated 48 hr	199 ± 2.9^ab^	21 ± 1.2^c^	129 ± 4.1^a^	45 ± 1.3^a^	105 ± 1.6^a^	285 ± 7.9^a^
Wheat flour	12–36¶	23¶				

Note

For flour from each type of pulse, means within column with different letters are significantly different (Tukey's test, *p* < .05). Regarding specific nutrients in raw flours, means with different capital letter are significantly different (Tukey's test, *p* < .05).

^†^ All values are mean ± *SD* of duplicate samples from duplicate trials (*n* = 4).

^‡^ Obtained by subtracting total H_4_folate before conversion from H_4_folate after conversion (Section 2.2.3).

^§^ Using a molecular weight of 445.4 for H_4_folate, 459.5 for 5‐CH_3_‐H_4_folate, 469.4 for 10‐HCO‐PteGlu, and 473.5 for 5‐HCO‐H_4_folate to calculate the sum of folate expressed as folic acid.

^¶^ Total choline is according to USDA (2018) and folate (expressed as total folate by microbiological analysis) is according to NFA (2018).

Lewis et al. (2014) report a total choline content (sum of esterified phospholipids) of 113–150 mg/100 g DM for flour from raw pea, lentil, black bean, red kidney bean, and pinto bean, which is 17%–28% lower than the values obtained in the present study. The lower total choline reported could be possibly caused by lack of standards for other choline‐contributing compounds, which are then not quantified (Hefni et al., 2018). The folate content of flours made from the raw pulses analyzed in this study (73–245 µg/100 g DM) was within the range (10–277 µg/100 g) reported by others (Rychlik, Englert, Kapfer, & Kirchhoff, 2007) for raw pea, lentils, white bean, kidney bean, and black bean. However, it was lower than values reported by the Swedish National Food Agency (NFA) for raw faba bean (423 µg/100 g) and white bean (488 µg/100 g) (NFA, 2018). The Swedish National Food Agency used a microbiological assay to measure total folate from all folate forms, which could explain the higher values (Hefni et al., 2010).

In treated pulse flours analyzed, the TDF content (15–24 g/100 g DM) increased by 11%–33% depending on the type of pulse and treatment. Similar content (17–31 g/100 g DM) and increase (9%–21%) were reported by others for flours from cooked white bean and pea (Aguilera, Martín‐Cabrejas, et al., 2009; Wang et al., 2008, 2010). This increase in TDF may be attributable to formation of retrograded resistant starch and insoluble by‐products from the Maillard reaction (Aguilera, Martín‐Cabrejas, et al., 2009).

After boiling, roasting, or germination, the RS content almost doubled in yellow pea flour and almost tripled in faba bean flour, but decreased by 18%–54% in gray pea flour and 80%–90% in white bean flour compared with the respective raw pulse flour (Table 2). Using the same analytical method, others found a twofold increase in RS in flours from roasted chickpea and boiled lentils, and an 88% decline in RS in black bean flour after cooking (Fabbri, Schacht, & Crosby, 2016; Wang, Hatcher, Toews, & Gawalko, 2009; Wang et al., 2010). There is no clear explanation for these conflicting results regarding RS in different types of pulses after processing, but according to Fabbri et al. (2016), they can be partly explained by different types of starch crystals and amylose/amylopectin ratios in pulses. Irrespective of the type of pulse, the RS content after treatment in the present study did not differ (*p* > .52). The RS content in flour from all pulses analyzed was within the range (3.5–4.2 g/100 g DM) reported for flours made from cooked black turle, cranberry, and red kidney beans (Wang et al., 2010).

A reduction of total choline content was observed after boiling in flour from faba bean (by 17%) and white bean (by 27%), but not in flours from peas. This is consistent with findings by others who reported a decrease (by 15%–22%) of total choline content after boiling of beans and peas due to leaching of free choline into the cooking medium (Lewis et al., 2014). Roasting had no effect (*p* > .11) on total choline content in any of the pulse flours (Table 3), which could be attributed to its stability toward heat (Ensminger, Ensminger, Konlande, & Robson, 1994). Germination did not affect (*p* > .41) total choline in yellow pea, gray pea, and faba bean flours, but led to a 15% reduction (*p* < .001) in white bean flour compared with the flours made from raw pulses (Table 3). No literature data on total choline content in pulses specifically after roasting and germination are available.

The almost doubled content of total choline in flours quantified in this study, as compared with reported data on pulse flours by others (Lewis et al., 2014), might be due to use of different analytical methods. Lewis et al. (2014) expressed total choline as the sum of five choline‐containing compounds. This might result in underestimation of the choline content as further choline‐containing compounds might be overlooked. In the present study, however, choline content was measured as a total after acid hydrolysis, which is expected to release all bound choline from phospholipids.

Depending on the type of pulse, boiling led to a 15%–32% decrease (*p* < .001) in folate content compared with the flour from raw pulses (Table 3). The Swedish National Food Agency and USDA report lower folate contents than in the present study, of around 81 µg/100 g in boiled white bean and 104 µg/100 g in boiled faba bean (NFA, 2018; USDA, 2018). These differences could be explained by the different varieties tested and analytical method used. Roasting increased (*p* < .001) the folate content by 1.1‐ to 1.2‐fold in white bean and pea flours in this study (Table 3). Hefni and Witthöft (2014) observed a 50% increase in folate content in faba bean and chickpea after soaking. Application of dry heat (such as roasting) might not cause leaching of folate from the pulse seeds, whereas boiling does (Delchier et al., 2013; Hefni & Witthöft, 2014), so roasting might lead to a net increase in folate content. Germination enhanced folate content by up to 1.2‐fold in white bean flour (*p* < .001) and 1.6‐ to 3.5‐fold in pea flours (*p* < .001) (Table 3). Shohag, Wei, and Yang (2012) also observed a 3.5‐ to 4.3‐fold increase in folate content (from 155–216 µg/100 g to 691–815 µg/100 g) in mungbean and soybean after 4 days of germination. This increase is probably caused by de novo synthesis of folate (Hefni & Witthöft, 2014; Jabrin, Ravanel, Gambonnet, Douce, & Rebeille, 2003).

Overall, treated bean flours had a higher nutrient content than treated pea flours (1.2‐fold higher TDF, up to 1.5‐fold higher total choline and 1.1‐ to 2.6‐fold higher folate). Flours from boiled, roasted, and germinated white bean had the highest content of TDF, RS, total choline, and folate, while flours from treated yellow pea had the lowest amounts of these nutrients. Compared with the commercial white wheat flour used as a reference, all treated pulse flours prepared in this study had 13‐ to 15‐fold higher RS, up to eightfold higher TDF, fourfold to 19‐fold higher total choline, and up to 13‐fold higher folate content.

### 3.3 Comparison of treatments and possible applications for treated pulse flours

The present study has shown that various treatments affected the functional properties and nutrient content of pulse flours differently. All treatments (boiling, roasting, and germination) increased water absorption capacity in pulse flours, which is in accordance with the literature (Acevedo, Thompson, González Foutel, Chaves, & Avanza, 2017; Aguilera et al., 2011; Benítez et al., 2013; Chitra, Singh, & Venkateswara Rao, 1996; Giami, 1993; Jogihalli, Singh, Kumar, & Sharanagat, 2017). Data from others also show an increase in oil absorption capacity in pulse flours after all treatments. The findings from the present study confirm this trend for flours prepared by roasting and germination, however, not for flours prepared by boiling. All treatment negatively affected parameters linked to emulsion, foaming, and gelling properties; this is in agreement with the previous work.

All treatments (boiling, roasting, and germination) enhanced the total dietary fiber content in pulse flours as also reported by others (Aguilera, Martín‐Cabrejas, et al., 2009; Benítez et al., 2013; Martín‐Cabrejas et al., 2003; Wang et al., 2008, 2010). Roasting and germination had no effect on total choline content but they increased folate content. Losses of total choline and folate were observed after boiling that is in agreement with others (Hefni & Witthöft, 2014; Lewis et al., 2014).

Flours from boiled, roasted, and germinated yellow pea, gray pea, faba bean, and white bean prepared in this study had high water and low oil absorption capacity. Thus, they could potentially be used in production of custards, sausages, doughs, and coatings for fried products. However, flours from treated gray pea had low emulsion and foaming properties, so their usefulness in products such as dressings, mayonnaise, cakes, and meat products might be limited. Furthermore, all treated flours from all pulse types had a high gelation value and are thus unsuitable for use as a sole thickening or gelling agent.

In theory, replacing 10% of refined wheat flour with treated pulse flour in a white bread formulation with initial TDF content of 3.5 g/100 g, total choline content of 15 mg/100 g, and folate content of 37.1 µg/100 g (NFA, 2018; Patterson et al., 2008) would significantly increase the TDF content (by 34%–58%), total choline (by 75%–133%), and the folate content (by between 7% (boiled yellow pea flour) and 67% (germinated white bean flour).

## 4 CONCLUSIONS

The functional properties and nutrient content of pulse‐based flours were found to be affected by treatment (boiling, roasting, and germination) and type of pulse. Irrespective of pulse type, boiling reduced the folate content markedly compared with roasting or germination. Thus roasted and germinated pulse flours could be used for bio‐fortification to enhance total dietary fiber, resistant starch, total choline, and folate content in for example, bakery goods, beverages, dressings, and coating batter for fried products. However, due to high gelation concentration value, use of treated pulse flours as a gelling or thickening agent may be limited.

## CONFLICT OF INTEREST

The authors have no conflict of interest to declare.

## ETHICAL APPROVAL

This study does not involve any human or animal testing.
